# Specially designed and CAD/CAM manufactured allogeneic bone blocks using for augmentation of a highly atrophic maxilla show a stable base for an all-on-six treatment concept: a case report

**DOI:** 10.1186/s40902-022-00351-9

**Published:** 2022-05-24

**Authors:** Florian Pfaffeneder-Mantai, Oliver Meller, Benedikt Schneider, Julius Bloch, Ditjon Bytyqi, Walter Sutter, Dritan Turhani

**Affiliations:** 1 Center for Oral and Maxillofacial Surgery, Department of Dentistry, Faculty of Medicine and Dentistry, Danube Private University, Steiner Landstraße 124, 3500 Krems, Austria; 2 Division for Chemistry and Physics of Materials, Department of Medicine, Faculty of Medicine and Dentistry, Danube Private University, Steiner Landstraße 124, 3500 Krems, Austria

**Keywords:** Bone block, Allograft, Maxillary atrophy, Maxillary augmentation, CAD/CAM

## Abstract

**Background:**

In terms of a highly atrophic maxilla, bone augmentation still remains very challenging. With the introduction of computer-aided design/computer-aided manufacturing (CAD/CAM) for allogeneic bone blocks, a new method for the treatment of bone deficiencies was created. This case report demonstrates the successful use of two specially designed and CAD/CAM manufactured allogeneic bone blocks for a full arch reconstruction of a highly atrophic maxilla with an all-on-six concept.

**Case presentation:**

We report the case of a 55-year-old male patient with a highly atrophic maxilla and severe bone volume deficiencies in horizontal and vertical lines. In order to treat the defects, the surgeon decided to use a combination of two allogeneic bone blocks and two sinus floor augmentations. The bone blocks were fabricated from the data of a cone beam computed tomography (CBCT) using CAD/CAM technology. After the insertion of the two bone blocks and a healing period of 7 months, six dental implants were placed in terms of an all-on-six concept. The loading of the implants took place after an additional healing time of 7 months with a screw-retained prosthetic construction and with a milled titanium framework with acrylic veneers.

**Conclusion:**

The presented procedure shows the importance of the precise design of CAD/CAM manufactured allogeneic bone blocks for the successful treatment of a highly atrophic maxilla. Proper soft-tissue management is one of the key factors to apply this method successfully.

## Background

The severe atrophic maxilla displays a surgical and prosthetic challenge due to the horizontal and vertical missing bone volumes when further implant placement is planned [1]. In the first 6 months after tooth extraction, horizontal bone loss of 3.96 mm and horizontal bone loss of 1.24 mm can be observed on average [2]. In order to regain sufficient bone volume, the following augmentation techniques can be used: bone blocks (autologous or allogeneic) [3, 4] interposition bone grafting techniques such as vertical splitting [5], horizontal sandwich technique or a Le-Fort-I osteotomy [6], guided bone regeneration with titanium meshes [7], or sinus floor elevations [8]. Other less invasive treatment options include the use of zygomatic implants [9], tilted implants, or short implants [10]. Although several treatment options for bone augmentation exist, the most effective method for achieving a sufficient horizontal bone dimension as well as a long-term implant and prosthesis survival is yet to be determined [11].

Bone resorptions are caused by the absence of mechanical load on the bone due to the loss of teeth which can be caused by trauma, periodontal diseases, caries, or infections with 40–60% of the ridge volume being lost in the first 3 years [12]. To determine the resorption of the alveolar ridge, Cordaro et al. [13] classified four different stages of bone loss for implants (quarter rule). Each stage is associated with a suitable treatment method. Stage one shows ¼ of bone loss of the facial/vestibular wall. Stage two shows the entire loss of the buccal wall and how a knife-edged ridge is formed. Stage three displays an overall height loss, while the buccal wall is partly intact. Lastly, stage four illustrates a fully resorbed and flat alveolar ridge. Stage one should be treated with a guided bone regeneration with simultaneous implantation, stage two with a bone block with simultaneous or delayed implantation, stage three with a bone block shell technique and later implantation, and stage four with a distraction osteogenesis.

Autogenous bone grafts are still considered the gold standard for evidently biological reasons (osteoconductive, osteoinductive, osteogenic) [14]. However, they show disadvantages such as postoperative morbidity, higher risk of neurological and vascular damage at the harvest site, limited bone availability, and bone resorption. Therefore, the use of allografts increased over the last years. Allografts can be divided into different types such as cortico-cancellous containing cortical layers or cancellous without cortical layers. They are available as cortical granules, cortical wedges, cortical chips, bone blocks, or cancellous powdered grafts [15]. The benefits of allografts are the decrease in postoperative morbidity, the possibility of individual block design with computer-aided design/computer-aided manufacturing (CAD/CAM) and lower resorption rates compared to autografts between the graft and the implant [16]. Moreover, in a recent meta-analysis, Troeltzsch et al. [14] showed that horizontal and vertical gains were higher for allogeneic bone blocks with weighted means between 3.7mm for xenogeneic and 4.6 ±1.4mm for allogeneic bone blocks. In order to minimize the risk of disease transmission, allografts can be divided into three groups according to their preparation: freshly frozen bone (FFBA), freeze-dried bone (FDBA), or demineralized freeze-dried bone allografts (DFDBA). Hence, the risk of transmission of the human immunodeficiency virus (HIV) is decreasing low, estimated at 1 in 1.6 million [17].

When Eufinger et al. showed the application of CAD/CAM manufactured onlay-blocks in-vitro in 1994, a new augmentation method was introduced [18]. Later, in another study, Schlee et al. [19] demonstrated horizontal and vertical ridge augmentations using CAD/CAM milled allogeneic bone blocks to treat posterior mandibular defects. In 2018, furthermore, Blume et al. successfully used CAD/CAM manufactured allogeneic bone blocks for bilateral maxillary augmentation [20].

As of today, CAD/CAM technology allows the individualized production of allogeneic bone blocks for complex alveolar ridge augmentation procedures. Various case reports successfully showed the accuracy and precision of these CAD/CAM manufactured bone blocks. Nevertheless, there are only a few reports about long-term results and survival rates [21]. Regarding their use for a full arch rehabilitation of the maxilla, the literature provides spare results [22].

The present case report describes the use of two custom CAD/CAM manufactured allogeneic bone blocks for an edentulous patient who suffered from a pronounced atrophic maxilla with a stage three class bone defect. It further shows the successful stabilization of dental implants in terms of an all-on-six concept with a screw-retained prosthesis on a titanium framework.

## Case presentation

**Fig. 1:**
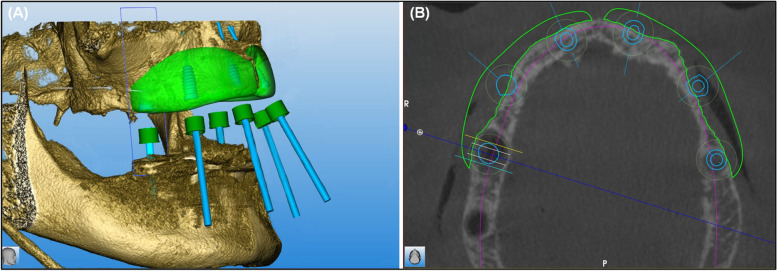
Digital planning based on a 3D X-ray image (DICOM file) for the design of the planned allogeneic bone blocks, including optimised subsequent implant position

**Fig. 2:**
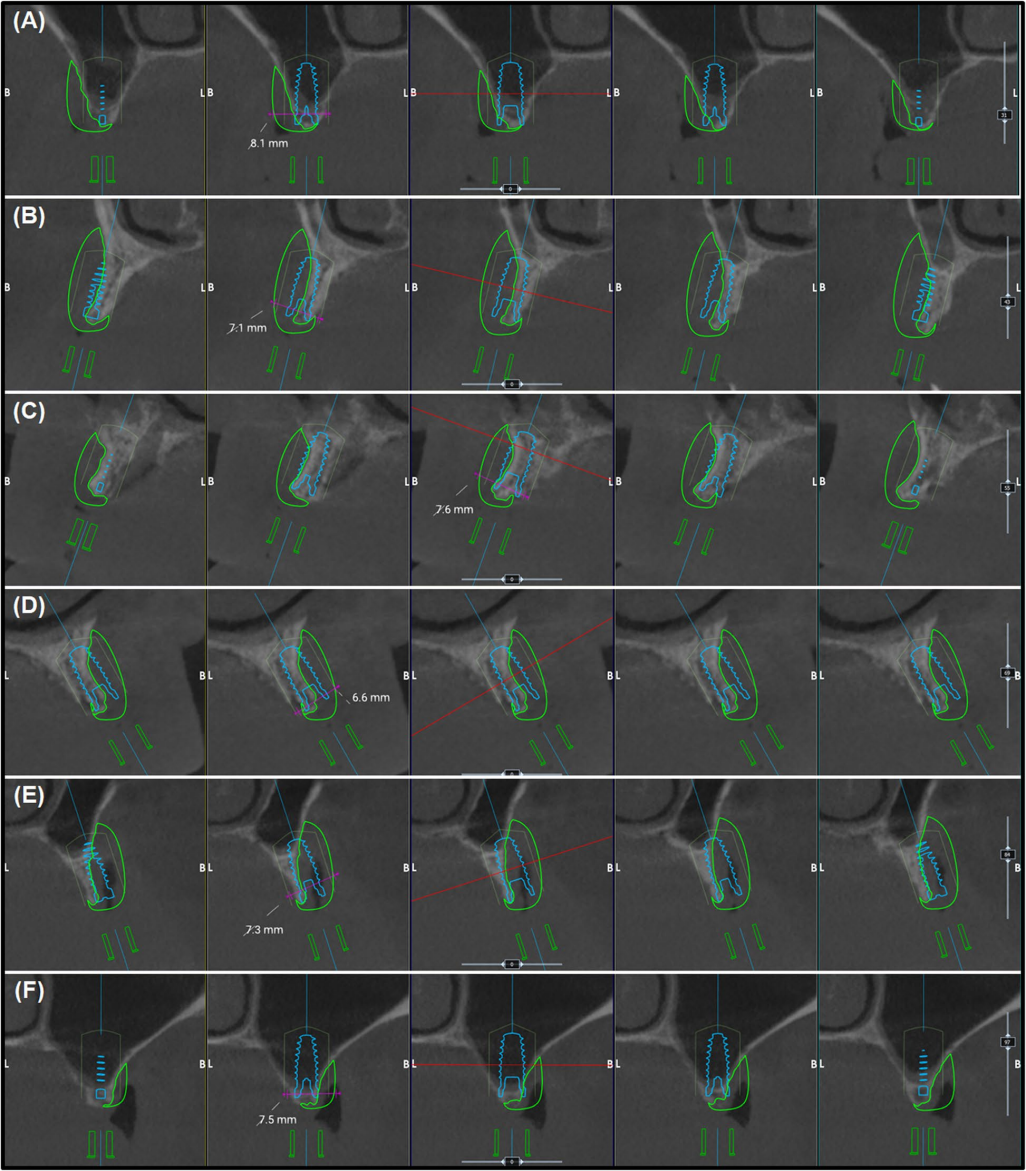
Digital planning of the special bone block design (in J-shaped) with implant placement position for the regions: **A** 16, **B** 14, **C** 12, **D** 22, **E** 24, and **F** 26

**Fig. 3:**
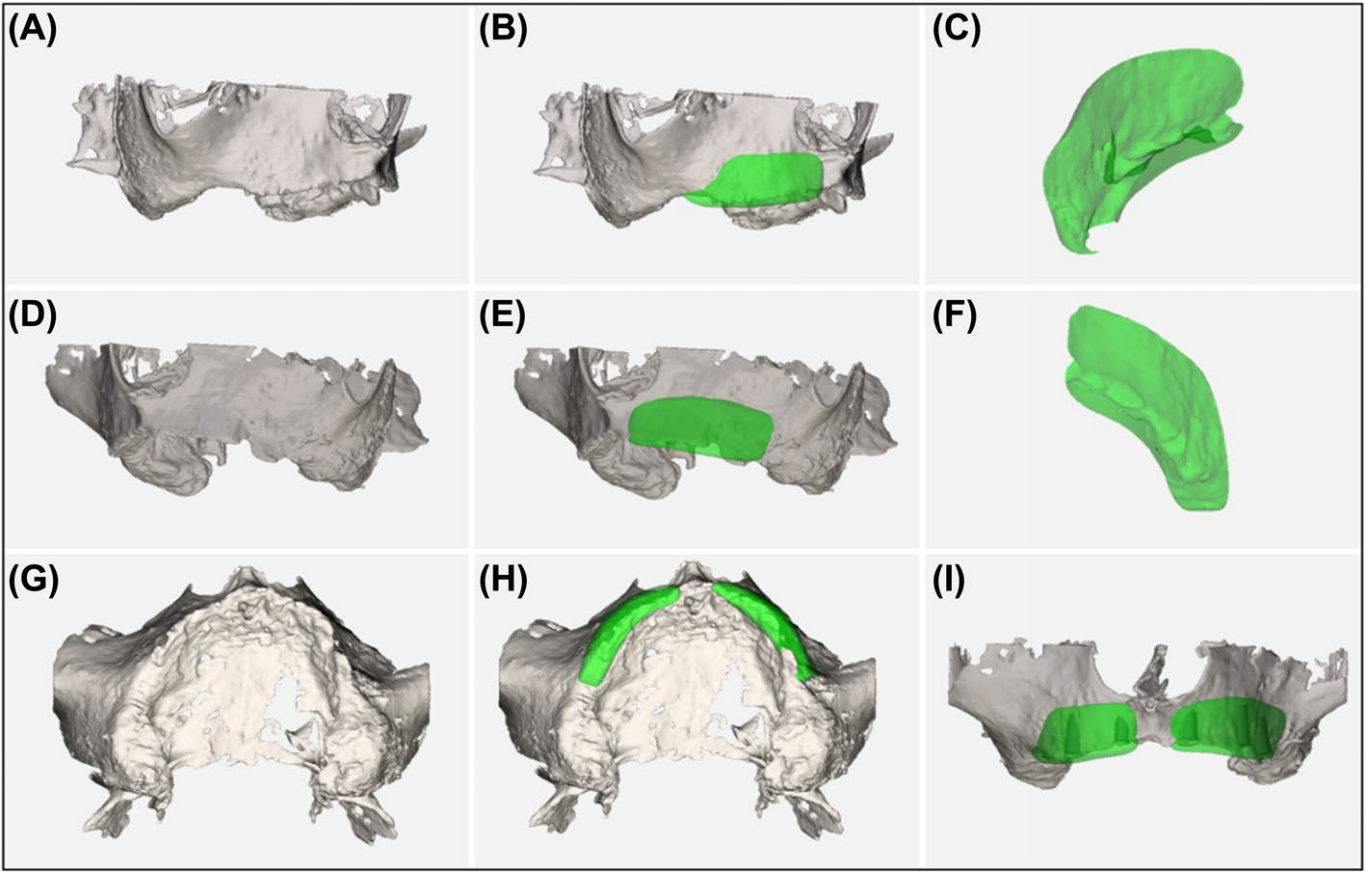
Planning and designing process of the two CAD/CAM manufactured allogeneic bone blocks to fit exactly on an accurate defect model (J-shaped design): **A**, **D**, and **G** Defect geometry; **B**, **E**, and **H** Allogeneic bone blocks on the defect model; **C** and **F** Allogeneic bone blocks; and **I** Allogeneic bone blocks, including optimized subsequent implant positions

We report the case of a 55-year-old male patient with a highly atrophic maxilla and the wish for further treatment in 2019. The patient had no allergies or diseases and was in good health. Due to the extensive bone volume losses (according to Terheyden bone loss classification stage three), the surgeon decided to use two CAD/CAM manufactured allogeneic bone blocks and two eternal sinus lifts to regain enough bone for the planned implantation. Therefore, during the first appointment, an intraoral photo status, an orthopantomography (OPT), and a cone beam computed tomography (CBCT) in DICOM format were taken (Fig. 1). The gained DICOM data was sent to the manufacturer (Zimmer Biomet Dental, USA) where the individual blocks were made (Fig. 2). To further enhance the fitting of the blocks on the alveolar ridge, the surgeon as well as the manufacturer designed the block with a special overlapping “J-shape-design” (Fig. 3). After the surgeon was satisfied with the block design, the manufacturer fabricated the two allogeneic bone blocks using (Puros® Allograft, Zimmer Biomet Dental, USA) CAD/CAM technology and further processed it with the Tutoplast® process [23]. The grafts were processed with alkaline, osmotic, oxidative, solvent, and irradiation treatment to eliminate the possibility of disease transmission without compromising its biological or mechanical properties.

After delivery of the allogeneic bone grafts as well as two three-dimensional models of the upper maxilla, the second appointment took place. The patient was given a 0.12% CHX mouth rinsing solution (GSK-Gebro Consumer Healthcare, Austria) and subsequently was locally anesthetized with Ultracain D-S forte with added epinephrine 1:100000 (Sanofi, France). An incision along the alveolar ridge of the maxilla was performed and a mucoperiosteal flap in both quadrants was created to allow a better overview for the later application of the bone blocks.

**Fig. 4:**
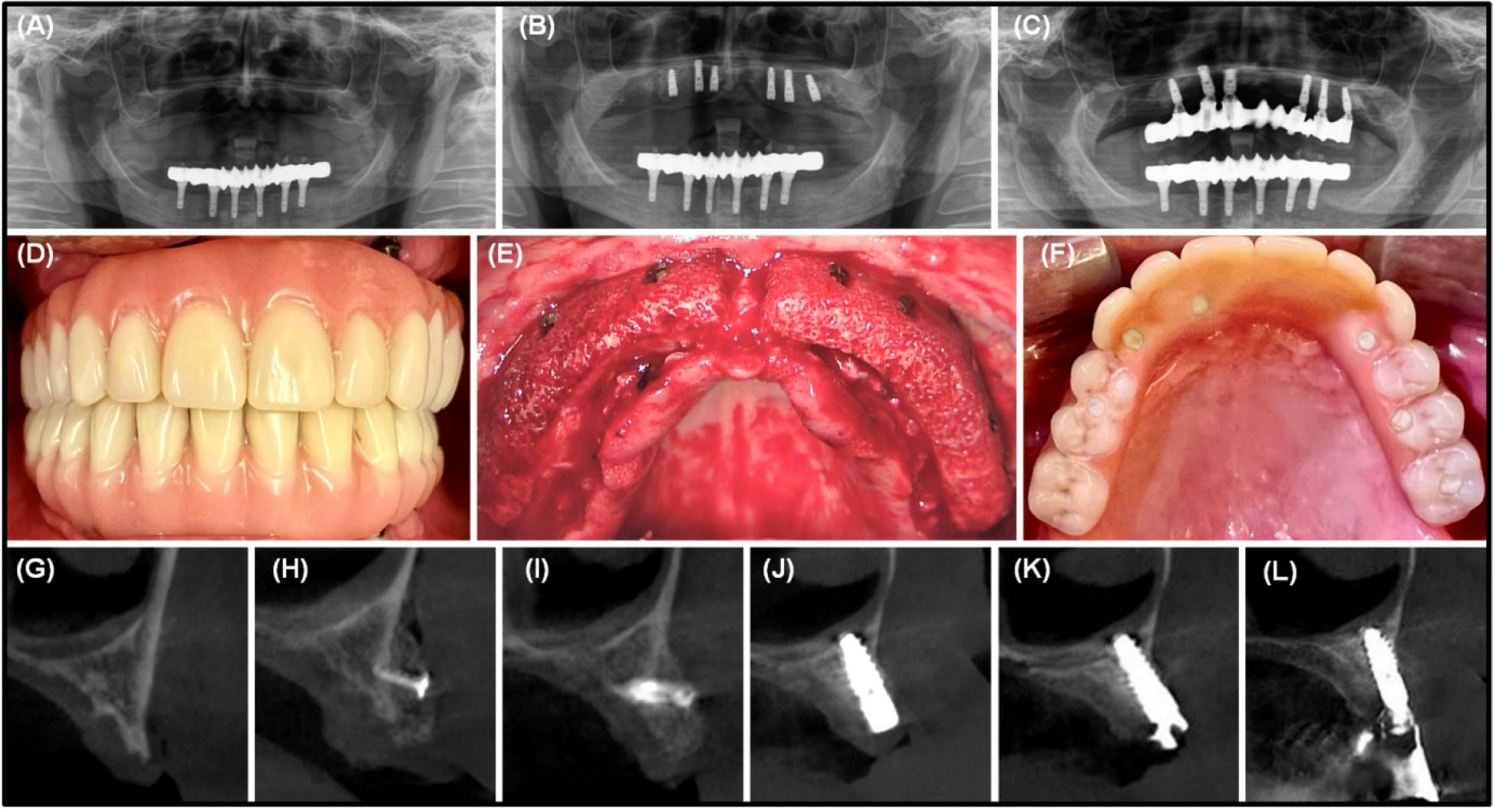
**A** OPT from the preoperative situation; **B** OPT after the implantation; **C** OPT shows the final screw-retained prosthetic construction; **D** clinical photo of the final rehabilitated maxilla (frontal view); **E** intraoperative image of two allogeneic individual 3D-bone blocks with simultaneous bilateral external sinus lifts for the augmentative rehabilitation of the upper jaw; **F** clinical photo of the finally rehabilitated upper jaw (occlusal view); **G**–**L** radiological chronology of augmentative, implantological, and prosthetic rehabilitation of a highly atrophic maxilla using combined therapy with external sinus lifts and 3D-milled bone blocks over a period of 19 months: **G** CBCT before augmentation (3 months); **H** CBCT after augmentation OP; **I** CBCT after augmentation (3 months); **J** CBCT after augmentation (12 months); **K** CBCT after augmentation (19 months); and **L** CBCT after augmentation (26 months)

Then, the surgeon performed two external sinus lifts in both quadrants of the maxilla in the molar and premolar regions. One sinus window on each side was created using the modified Caldwell-Luc approach, and then, the Schneiderian membranes were carefully elevated with a sinus curette. The surgeon filled the sinus cavities with cancellous allogeneic bone particulates (Puros® Cancellous Particulate Allograft, Zimmer Biomet Dental, USA). After that, the first try-in of the CAD/CAM manufactured allogeneic bone blocks was performed. The two blocks seemed to fit the defect areas perfectly and were therefore considered suitable for further placement. The blocks were fixated to the alveolar ridge using osteosynthesis screws (Craniofacial Modular Fixation System, DePuy Synthes, Johnson & Johnson, USA) which were made out of corrosion-resistant medical steel (Fig. 4). Then, the transition between the bone blocks and the alveolar ridge was smoothed and a bovine pericardial membrane (CopiOs® Zimmer Biomet Dental, USA) was subsequently placed over the augmented area in sense of a guided bone regeneration. Finally, the mucoperiosteal flap was readapted and closed with non-resorbable polypropylene sutures 4-0 (Ethicon, Johnson & Johnson, USA) with a single button and mattress sutures in a saliva-proof and tension-free manner.

After a healing period of 7 months following the augmentation, the third appointment for further implantation took place. With the same procedure as last time, the patient was anesthetized, a mucoperiosteal flap was created and six implants (Straumann® BLX, Switzerland) in the sense of all-on-six were placed in regions 11, 13, 15, 21, 23, and 25. The implant diameters and lengths were 3.75×10mm for regions 11, 13, 21, 23, and 3.75×12mm for regions 15 and 25. The implants showed proper primary stability (>35 Ncm). The mucoperiosteal flap was sutured tension-free with non-resorbable polypropylene sutures 4-0 (Ethicon, Johnson & Johnson, USA). After the operation, an OPT was taken to show the position of the implant placement in coordination with the digitally generated bone blocks (Fig. 4).

After an additional healing time of 7 months, the patient had an appointment for impression-taking of the situation. The impressions were used to create the definitive restoration of the maxilla. In the meantime, the patient was provided with a removable complete denture for the maxilla and was monitored every 3 months to prevent the risk of pressure marks of the denture.

Finally, 19 months after the bilateral bone block augmentation, the patient was provided with a screw-retained prosthetic construction with a milled titanium framework with acrylic veneers (Fig. 4).

## Discussion

Nowadays, autologous bone transplants are still considered the gold standard. However, allogeneic bone grafts show a lesser risk of infections, postoperative morbidity, and unlimited bone availability. Although the clinical data shows excellent survival rates for allogeneic bone blocks ranging from 93.7 to 100.0%, the application of the allogeneic bone blocks is assumed to be more technique-sensitive [24, 25]. Looking at the bone gain of allogeneic bone blocks, the literature provides several studies and meta-analysis with horizontal and vertical bone gains of 4.6±1.4 mm on average. Considering the loss of horizontal and vertical dimensions, allogeneic bone blocks showed better results than autogenous blocks with 0.4±0.5mm horizontal and 0.6±1.0 mm vertical for allogeneic vs. 1.2±3.4 horizontal and 2.9±1.9 mm vertical for autogenous blocks [14].

Nonetheless, allogeneic bone blocks are associated with common complications like wound dehiscence resulting in membrane exposure, incision line opening, and bone block exposure. These drawbacks are not caused by the allogeneic blocks itself, but rather due to poorly soft-tissue management which was confirmed in a study by Chaushu et al. where 137 complications after allogeneic bone block augmentation were analyzed [26]. Further, a negative outcome like the loss of an allogeneic bone block can be induced by a thin gingiva type, a pre-existing disease, or bad oral hygiene [27]. If the bone block is exposed, it does not always has to be removed in its entirety. An extensive debridement of the infected area can still provide enough volume for later implantation even if the block was partially lost. In a recent systematic review by Starch-Jensen et al. [28], no difference was found in the treatment outcome with implants after ridge augmentation with autogenous versus allogeneic bone blocks. However, fewer complications were reported when using autogenous bone blocks [28].

According to the S3-Guideline by the German association of oral implantology (DGI), a full arch reconstruction of the maxilla should at least be realized with four implants either with a fixed or removable prosthesis. When using an all-on-six concept with a fixed prosthesis the 10-year survival rates vary in several studies between 94 and 96%. Therefore, it is considered to be a safe treatment solution [29–31].

In order to achieve enough insertion torque and stability, six implants (Straumann® BLX, Switzerland) were used. The implants incorporate a double-thread design with bi-directional cutting threads and a reduced neck diameter. Therefore, they are believed to apply lesser pressure on the crestal bone and were able to show proper primary stability in some case reports [32, 33].

With the introduction of CAD/CAM technology, individualized bone blocks can be designed from the data of CBCT scans. The manual adaption of the customized block is mostly obsolete due to the accuracy of the technology, which reduces the risk of contamination. The blocks also show a perfect fit to the donor side [34]. The presented case report highlights the advantages of these CAD/CAM fabricated bone blocks in a highly atrophic maxilla. The two allogeneic bone blocks fitted perfectly into the defects of the alveolar ridge and showed no complications due to proper soft-tissue management as well as proper saliva-tight and tension-free covering. These points were crucial for the following healing period, the overall volume gain of the bone blocks, and for further implantation. With an overall treatment time of 19 months, the procedure remains time-consuming and challenging for the practitioner.

## Conclusion

This case report is one of the first that demonstrates the treatment and regeneration of a highly atrophic maxilla with the implementation of two CAD/CAM manufactured allogeneic bone blocks. We successfully showed that the two CAD/CAM manufactured bone blocks led to a sufficient full arch reconstruction as well as a successful all-on-six implantation. Further, allogeneic bone blocks represent a great and less invasive alternative to other augmentation techniques such as an interposition osteotomy like a Le Fort-I or maxillary advancement with an iliac crest. To predict the long-term survival rates of CAD/CAM fabricated allogeneic bone blocks used for a full arch rehabilitation, further research is needed.

## Data Availability

Data sharing is not applicable to this article as no datasets were generated or analyzed during the current case report.

## Abbreviations

CAD/CAM: Computer-aided design/computer-aided manufacturing
CBCT: Cone beam computed tomography
CHX: Chlorhexidine
DFDBA: Demineralized freeze-dried bone allografts
DGI: German association of oral implantology
DICOM: Digital Imaging and Communications in Medicine
FDBA: Freshly frozen bone
FFBA: Freeze-dried bone
HIV: Human immunodeficiency viruses
OPT: Orthopantomography
